# Dam prepartum skeletal muscle reserves and supplementation with branched-chain volatile fatty acids during late gestation influence calf birth weight and calf muscle metabolic activity

**DOI:** 10.3168/jdsc.2024-0581

**Published:** 2025-03-03

**Authors:** Linda M. Beckett, Brianna Gast, Evy Tobolski, Lauren Jones, Kyrstin Gouveia, Yu Han-Hallett, Theresa Casey, Jacquelyn P. Boerman

**Affiliations:** 1 Department of Animal Sciences, Purdue University, West Lafayette, IN 47907; 2 Bindley Bioscience Center, Purdue University, West Lafayette, IN 47907

## Abstract

Dairy cattle mobilize skeletal muscle in the periparturient period to close energy and metabolizable protein gaps due to the high demands of the growing fetus and milk production. The objective of this study was to determine if the amount of dam prepartum muscle reserves and branched-chain volatile fatty acids (BCVFA) supplementation affected calf birth weight, calf circulating AA, calf semitendinosus muscle metabolic activity, and colostrum composition. We hypothesized that calves born to dams with higher prepartum muscle reserves and supplemented with BCVFA would be heavier, have greater muscle mass as determined by circulating creatinine, and have higher muscle metabolic activity compared with calves born to low-muscle dams not supplemented with BCVFA. At 42 d before expected calving (BEC), the depth of the longissimus dorsi muscle was measured via ultrasound and cows were classified as high muscle (HM; >4.6 cm) or low muscle (LM; ≤4.6 cm) and then randomly assigned to either control (CON) diet, which consisted of soy hulls or calcium salt products of BCVFA that were top dressed from 42 d BEC to parturition. The final study design was a 2 × 2 factorial of muscle reserves and BCVFA supplementation, resulting in LM-CON (n = 8), LM-BCVFA (n = 10), HM-CON (n = 12), and HM-BCVFA (n = 10). Calves were weighed and colostrum was fed to calves at 10% of birth BW and a second dose at 5% of birth BW. At 24 h postnatal, a jugular blood sample and biopsy from the semitendinosus muscle was collected. Metabolic activity of the semitendinosus muscle was measured with a resazurin based assay. Calves born to HM cows were heavier at birth and had higher circulating creatinine and higher muscle metabolic activity, but dam BCVFA supplementation did not affect any of these parameters. Neither maternal muscle reserves nor BCVFA affected calf muscle mTOR abundance or phosphorylation state, although LM-CON calves tended to have lower phosphorylated mTOR than other groups. The greater birth weight, greater circulating creatinine, and higher muscle metabolic activity of calves born to HM cows suggest that maternal muscle reserves influence fetal growth and muscle mass as well as muscle metabolic phenotype.

## Highlights

• In utero exposure to high-muscle phenotype led to heavier calves.
• In utero exposure to high-muscle phenotype led to calves with more muscle mass.
• Calves born to low-muscle dams had increased circulating AA at 24 hours postnatal.
• BCVFA fed to dams had minimal impacts on calf measures.

## Graphical Abstract

**Figure:**
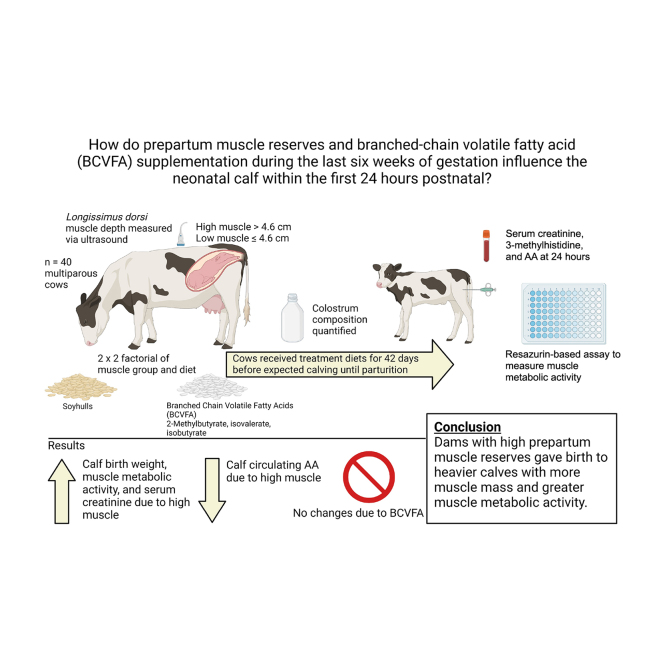
**Summary:** Forty prepartum dairy cows were stratified by longissimus dorsi muscle depth and fed branched-chain volatile fatty acids (BCVFA) 42 days before expected calving. Calf parameters, including birth weight, blood metabolites, and muscle metabolic activity, were collected at 24 hours postnatal. Calves born to high-muscle dams had greater birth weight, muscle mass, and muscle metabolic activity compared with calves born to low-muscle dams. Calves born to low-muscle dams had higher circulating amino acids (AA) at 24 hours postnatal. Supplementation of BCVFA had minimal impacts on colostrum composition or calf parameters. In utero exposure to a high-muscle phenotype had the greatest impact on calf parameters compared with BCVFA supplementation.

Growth of the calf fetus increases exponentially during the last 30 d of gestation (Bauman and Currie, 1980) and the fetus relies on glucose and AA from the dam to support fetal growth (Bell, 1995). Despite glucose being the primary energy source for the growing fetus, AA requirements in late gestation are 3 times higher than CP requirements because AA are rapidly catabolized by the developing fetus. Only 32% of metabolizable AA available to the fetus are deposited in fetal tissues (Bell et al., 1992; Bell, 1995), further underscoring the need to better understand maternal nutrient supply to the calf.

Circulating levels of creatinine can be used to estimate relative muscle mass, with higher creatinine indicating greater mass (Megahed et al., 2019), whereas circulating 3-methylhistidine (**3-MH**) is an indicator of muscle mobilization. The ratio of the concentration of 3-MH to creatinine is used to estimate muscle turnover (Pires et al., 2013; Sadri et al., 2023). Previous studies in our laboratory found that the extent dairy cows mobilize muscle, as estimated by change in depth of longissimus dorsi (**LD**) muscle from 42 d before expected calving (**BEC**) to early lactation (30 DIM), varied by the amount of LD present at 6 wk prepartum (McCabe et al., 2021). Cows categorized as high muscle (**HM**) at the start of the study maintained muscle prepartum, whereas low-muscle (**LM**) cows accreted muscle, and calves from HM dams were heavier (McCabe et al., 2021).

Branched-chain volatile fatty acids (**BCVFA**) supplements for cows were developed to support microbial protein supply and in turn support metabolizable protein (Mitchell et al., 2023a) and fiber digestibility (Firkins, 2010; Roman-Garcia et al., 2021). Branched-chain VFA consist of 2-methylbutyrate, isobutyrate, and isovalerate. In the rumen, branched-chain AA (**BCAA**) Ile, Leu, and Val can be deaminated to produce BCVFA. Alternatively, BCVFA can be transaminated into keto acids that are decarboxylated to form BCAA (Mitchell et al., 2023b). Supplementing weaned dairy calves with BCVFA increased calf ADG, feed efficiency, and total-tract digestibility compared with calves not supplemented with BCVFA (Liu et al., 2020). Male Holstein calves supplemented with BCVFA at the rate of 3.5 and 4 g/kg DM demonstrated increased DMI, ADG, nutrient digestibility, circulating glucose, and hepatic expression of mTOR compared with no BCVFA supplementation (Liu et al., 2019). Currently, little is known regarding how BCVFA supplementation during late gestation affects the fetus and colostrum composition. Therefore, the objective of this study was to evaluate how prepartum muscle reserves and BCVFA supplementation affect calf size, muscle metabolic activity, circulating metabolites, and colostrum composition. The overall hypothesis of this study was that dam prepartum muscle reserves influence both fetal growth in utero and colostrum composition. Specifically, dams with more prepartum skeletal muscle reserves and supplemented with BCVFA will produce calves with more muscle mass and increased muscle metabolic activity, as well as increased protein content of colostrum with altered AA composition compared with their control-fed and low-muscle reserve counterparts.

The experimental protocols were reviewed and approved by Purdue University Animal Care and Use Committee (protocol no. 2109002197) before beginning the study. Results of POWER procedure analysis in SAS 9.4 (SAS Institute, Cary, NC) using expected variation in calf birth BW as the primary variable indicated 8 animals per treatment was adequate to achieve 90% power. All cows were housed at the Purdue University Dairy Research and Education Center (West Lafayette, IN) in tiestalls and were released twice a day at 0600 h and 1600 h to exercise. This experiment was part of a larger study (n = 48 cows) reported by Gouveia et al. (2024) aimed at understanding the effects of muscle reserves and BCVFA supplementation on multiparous cow production performance. A subset of 40 late-gestation multiparous dairy cows was used in the current study. Cows were assigned to treatments in a 2 × 2 factorial design. At 42 d BEC, depth of the LD muscle was assessed using ultrasound with an Aloka SSD-500 ultrasound machine (Aloka, Wallingford, CT). One individual captured the ultrasound images of the LD muscle depth in triplicate. Muscle depth was quantified using ImageJ software (version 1.54; National Institutes of Health, Bethesda, MD) and 3 ultrasound images were averaged by the same individual. Animals with muscle depth ≤4.6 cm were assigned to the LM group (n = 18) and cows with muscle depth >4.6 cm were assigned to the HM group (n = 22). Cows were then randomly allocated to one of 2 dietary treatments: control (CON; n = 20) or BCVFA (n = 20). The final treatment groups were LM-CON (n = 8), LM-BCVFA (n = 10), HM-CON (n = 12), and HM-BCVFA (n = 10). As reported in Gouveia et al. (2024), both diets had the same ingredients and nutrient composition, except that 73.0 g/d DM of soy hulls were top dressed on the TMR for the CON diet, and BCVFA calcium salt products supplied by Zinpro Corporation (Eden Prairie, MN) containing 19.6 g/d 2-methylbutyrate DM, 19.4 g/d isovalerate DM, and 39.1 g/d isobutyrate DM were top dressed for the BCVFA diet. The BCVFA calcium salt products were analyzed by Zinpro Corporation to determine BCVFA concentrations using the procedure described by Roman-Garcia et al. (2021). Cows received the respective dietary treatments from 42 d BEC until parturition. Cows were fed once daily at 0900 h for ad libitum intake, which was determined by weighing residual feed from the previous day and feeding 10% above this level. Cows had free access to water.

Beginning at 7 d BEC, cows were checked every 4 h for signs of parturition. Upon calving, cows were moved to a locking headgate to restrain for milking with a portable milking unit (Chore Boy, Hamby Dairy Supply, Maysville, MO) to collect colostrum within 4 h of parturition. We preserved 8 mL of colostrum in 1-mL aliquots at −20°C for future compositional analysis. Within 4 h of birth, calves were sexed and weighed, and then bottle fed their dam's fresh colostrum at 15% of birth BW with 10% of the total amount being fed within 4 h of birth. The fresh colostrum was then frozen and the remaining 5% of the 15% allotment was fed 12 h after the first dose. At 24 h postnatal, hip width, hip height, and wither height were measured, blood was sampled via jugular venipuncture, and a biopsy of the semitendinosus muscle was taken as described in Beckett et al. (2019).

Colostrum was analyzed for fat using the creamatocrit protocol (Lucas et al., 1978). Colostrum protein percentage was quantified using the bicinchoninic acid assay (ThermoFisher Scientific, Waltham, MA) following manufacturer's instructions and diluting the samples 1:500 in 1× PBS before analysis. Serum and plasma were isolated from whole blood and used for analysis of immunocrit, glucose, AA, 3-MH, and creatinine. Immunocrit was quantified using the protocol by Vallet et al. (2013). Glucose was quantified using a Wako Glucose AutoKit (Fujifilm Wako Chemicals). Free AA concentration (ng/μL) in colostrum and serum of neonates was measured using liquid chromatography tandem MS (**LC-MS/MS**) following the extraction protocol and LC-MS/MS parameters in Bitsie et al. (2021). Serum concentration of 3-MH and creatinine was also measured using LC-MS/MS as described by Gouveia et al. (2024).

Muscle biopsies of the semitendinosus were placed in 30 mL of Dulbecco's Modified Eagle Medium (**DMEM**; Fisher Scientific; low glucose [5 m*M*], pyruvate) with 1% antibiotic antimycotic (**ABAM**; Sigma Aldrich) solution containing 10,000 units penicillin, 10 mg streptomycin, and 250 ug/mL amphotericin B to be transported back to the laboratory on ice. Muscle metabolic activity was measured in triplicate with ∼20 mg of tissue and using a resazurin based assay as described by Beckett et al. (2019). Wells were preloaded with 200 μL of DMEM, 1% ABAM, and 1.6% alamarBlue (Molecular Probes alamarBlue Cell Viability Reagent, Fisher Scientific). Fluorescence was read at an excitation of 530 nm and an emission of 590 nm on a Tecan Spark 10M multimode plate reader (Tecan, Zürich, Switzerland) every 15 min for 1 h and stored covered with aluminum foil at 37°C between readings. A wet weight was collected after the readings were complete. The relative metabolic activity assay was evaluated first with the fixed effect of time (0, 15, 30, 45, and 60 min) that fluorescence was measured. We found no effect of time on relative metabolic activity, and data were therefore averaged to obtain one value per calf.

Approximately 80 mg of muscle tissue was collected, then snap frozen in liquid nitrogen for measuring mTOR and phospho-mTOR abundance using a phospho-mTOR and total mTOR ELISA kit (ab279869 Abcam, Cambridge, UK). The manufacturer's protocol was followed, using ∼80 mg of tissue homogenized in 1× cell lysis buffer from the mTOR ELISA kit for 30 s using a Fisherbrand Homogenizer 150 (Fisher Scientific, Waltham, MA). Samples were diluted 1:20 in 1× assay buffer, and duplicate wells of each sample were run for both pan-mTOR (unphosphorylated mTOR) and phospho-mTOR. The absorbance of samples was read at 450 nm using a Tecan Spark 10M multimode plate reader (Tecan, Zürich, Switzerland). Data were expressed as absorbance at 450 nm · g of tissue^−1^.

All data were analyzed using the MIXED procedure in SAS 9.4 (SAS, Cary, NC) following a check for normal distribution using the PROC Univariate procedure with the Shapiro-Wilk test *P*-value evaluated due to a sample size less than 50. Outliers were evaluated using the “influence” command in SAS, which outputs Cook's *D* values. A Cook's *D* value above 10/n (Cook, 1977) was considered an outlier and removed from the respective dataset. Tukey's honestly significant difference post hoc test was used to compare the effect of muscle group (LM vs. HM), dietary treatment (CON vs. BCVFA), and the interaction of muscle group and dietary treatment. The BW model also included the fixed effect of calf sex. Treatment means were determined to be different when *P* ≤ 0.05 and tended to be different when 0.05 < *P* ≤ 0.10. Pearson correlation coefficient analysis was employed using the PROC CORR procedure in SAS 9.4 to compare muscle depth at 42 d BEC to calf and colostrum parameters. A significant correlation was determined to be present when *P* < 0.05 and tended to occur when 0.05 ≤ *P* ≤ 0.10.

Dam muscle reserves and BCVFA treatment did not affect colostrum fat or protein concentration (Table 1). In the larger study, we found HM reserves increased milk yield but had no effect on milk fat or protein concentration, whereas BCVFA supplementation decreased fat concentration by 0.3% over the first 30 DIM with no effect on milk fat yield (Gouveia et al., 2024). The protein and fat profile of colostrum is distinct from mature milk, with colostrum containing a high level of immunoglobulins, which contribute to protein concentration (Playford and Weiser, 2021). Factors that affect mature milk composition appear to have less of a direct effect on colostrum composition.

**Table 1:** Colostrum fat, protein, and AA concentration from cows stratified by low muscle (LM) and high muscle (HM) or fed a control (CON) diet or a diet supplemented with branched-chain volatile fatty acids (BCVFA)^1^

Colostrum component	LM-CON	LM-BCVFA	HM-CON	HM-BCVFA	SEM	Muscle	Diet	Muscle × diet	Correlation with dam muscle depth at 42 d BEC	
									r-value	*P*-value
Fat, %	8.05	8.40	8.24	8.01	1.10	0.93	0.96	0.80	−0.09	0.59
Protein, %	35.2	28.9	32.9	33.9	2.47	0.58	0.28	0.14	0.15	0.36
AA, ng/μL										
Ala	7.39	6.96	7.35	8.78	0.75	0.24	0.51	0.23	0.29	0.06
Arg	1.34	0.98	0.72	0.78	0.24	0.09	0.53	0.38	−0.34	0.04
Asn	0.18^ab^	0.15^ab^	0.13^b^	0.23^a^	0.03	0.62	0.22	0.05	0.17	0.29
Asp	0.47	0.34	0.31	0.48	0.09	0.91	0.82	0.11	−0.007	0.96
Cys	0.08	0.07	0.09	0.19	0.03	0.08	0.17	0.13	0.27	0.09
Gln	0.31^a^	0.13^b^	0.20^ab^	0.19^ab^	0.04	0.51	0.03	0.04	−0.19	0.27
Glu	13.6	16.0	12.2	17.0	2.20	0.92	0.10	0.57	0.07	0.60
Gly	3.63	4.34	3.63	4.81	0.69	0.73	0.17	0.72	0.21	0.19
His	0.41	0.27	0.27	0.33	0.06	0.57	0.57	0.13	−0.27	0.12
Ile	2.66^a^	1.65^ab^	0.90^b^	1.22^ab^	0.56	0.03	0.49	0.19	−0.42	0.01
Leu	1.17	0.59	0.70	0.83	0.20	0.57	0.28	0.09	−0.18	0.29
Lys	0.80	0.65	0.61	0.80	0.16	0.92	0.92	0.33	−0.24	0.15
Met	0.22	0.30	0.43	0.23	0.10	0.49	0.50	0.13	0.15	0.37
Phe	0.45	0.60	0.52	0.40	0.14	0.66	0.93	0.34	−0.30	0.08
Pro	12.1	11.8	12.7	15.8	2.28	0.32	0.53	0.45	0.14	0.40
Ser	0.56	0.59	0.58	0.74	0.07	0.26	0.20	0.40	0.21	0.21
Thr	0.51	0.37	0.40	0.53	0.06	0.68	0.97	0.04	−0.001	0.99
Trp	0.14	0.16	0.18	0.13	0.04	0.96	0.80	0.45	−0.13	0.43
Tyr	1.56	2.59	3.47	1.15	0.70	0.75	0.38	0.03	0.03	0.86
Val	19.6	15.8	14.9	18.3	4.70	0.83	0.97	0.47	−0.16	0.36

a,b Values without a common superscript letter differ (*P* ≤ 0.05) when using a Tukey adjustment for mean separation.

1 Treatment means or correlation relationships were determined to be different when *P* ≤ 0.05 and tended to be different when 0.05 < *P* ≤ 0.10. Data are reported as LSM.

Colostrum Arg, Cys, and Ile from dams in the HM group were lower compared with LM dams (*P* ≤ 0.09). We found interactions of muscle by diet (*P* ≤ 0.09) for colostrum Asn, Leu, and Thr; HM-BCVFA had increased concentrations, whereas LM-CON had the lowest concentrations. The concentration of Gln decreased with LM-BCVFA (muscle × diet; *P* = 0.04). and the concentration of Tyr increased in LM-BCVFA and HM-CON (muscle × diet; *P* = 0.03). Colostrum Arg, Ile, Ala, and Phe concentrations negatively correlated with dam muscle depth (*P* < 0.10) and colostrum Cys positively correlated with dam muscle depth (*P* = 0.09). There are 2 possible explanations for increased Leu concentration in HM-BCVFA: BCVFA conversion to BCAA in the rumen, or bacteria incapable of converting isovalerate to Leu via the isopropylmalate pathway due to the lack of the isopropylmalate synthase may shunt isovalerate to Leu synthesis in the rumen (Allison et al., 1984), possibly changing the ruminal pool size of Leu and potentially altering the supply of Leu to the rest of the system. The LM animals accreted more muscle prepartum (Gouveia et al., 2024), suggesting circulating AA would be used for muscle protein synthesis rather than being partitioned toward colostrum; thus, increased colostrum Ile was unexpected. Treatments may not directly explain changes in colostrum AA because AA can interconvert into other AA through aminotransferases, which can influence the pool sizes of different AA available to colostrum.

We found an effect (*P* < 0.01) of calf sex on birth BW, with bull calves (n = 12) weighing 6.36 kg more than heifer calves (n = 28). Calves born to HM cows weighed 3.63 kg more than those born to LM cows (*P* = 0.01; Figure 1A). Bull calves were equally distributed between all treatments due to random occurrence (n = 3 per treatment); therefore, treatment responses are not confounded by calf sex. Calf birth BW positively correlated with dam muscle depth at 42 d BEC (r = 0.35; *P* = 0.02). The finding that calves born to high-muscle dams were heavier is consistent with our previous findings (McCabe et al., 2021). Calves born to HM cows had higher circulating creatinine concentrations (*P* = 0.10), which supports the potential that greater calf weight was due to greater muscle mass. Supplementation with BCVFA did not affect calf birth body weight. Calf hip height, hip width, and wither height at 24 h postnatal were not affected by dam muscle reserves nor BCVFA supplementation. We found no effect of dam muscle reserves, BCVFA, or their interaction on calf immunocrit, glucose, 3-MH, or the ratio of 3-MH to creatinine, nor any significant correlations with dam muscle depth at 42 d BEC. The lack of effects on circulating nutrients may reflect the 12-h fast of calves before blood sampling.

**Figure 1:**
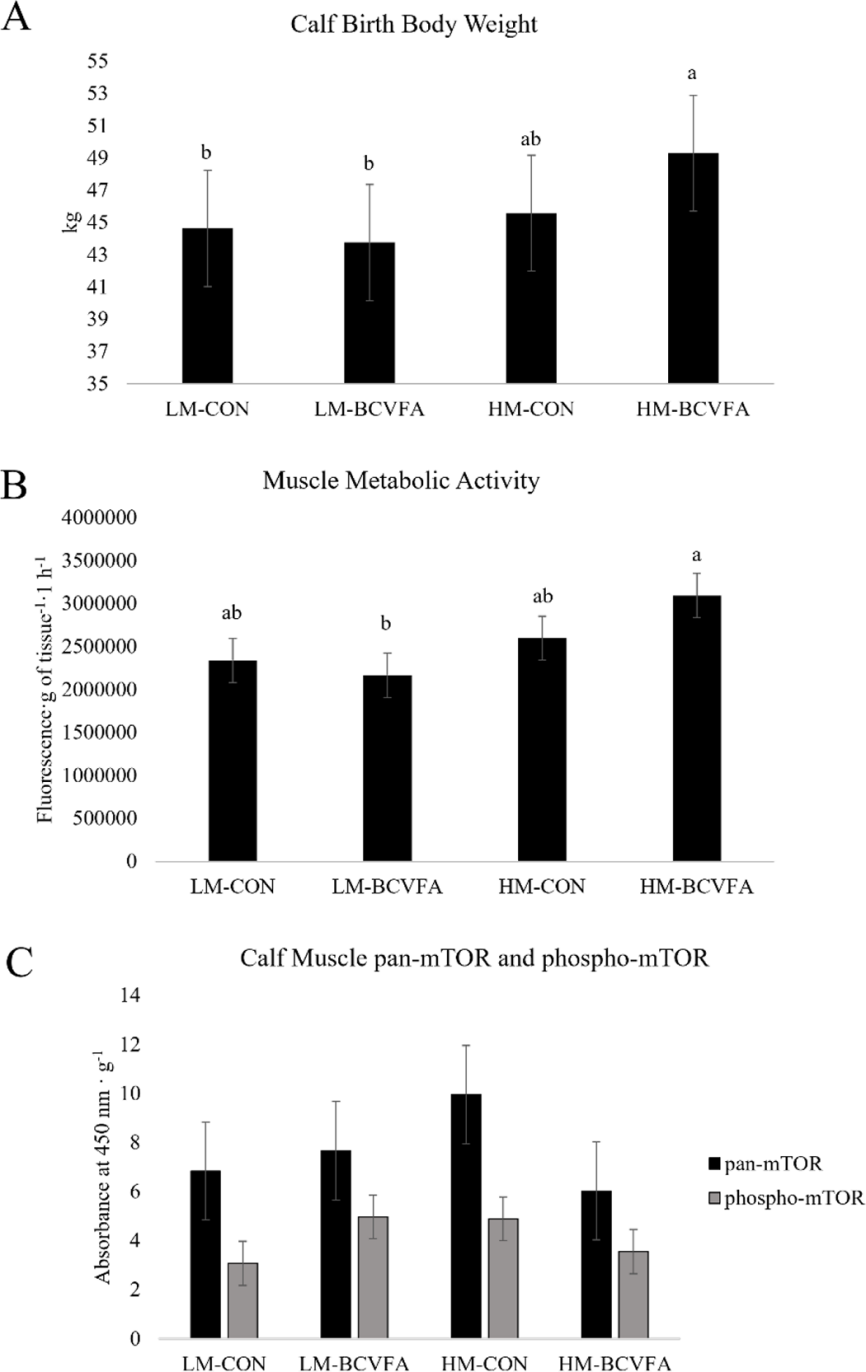
Calf birth body weight (A), semitendinosus muscle metabolic activity (B), and semitendinosus muscle phosphorylated mTOR and pan-mTOR measures (C) from calves born to dams stratified by low muscle (LM) and high muscle (HM) and fed a control (CON) diet or a diet supplemented with branched-chain volatile fatty acids (BCVFA). Treatment means were determined to be different when *P* ≤ 0.05 and tended to be different when 0.05 < *P* ≤ 0.10. Data are reported as LSM ± SEM, and values without a common lowercase letter differ (*P* ≤ 0.05) when using a Tukey adjustment for mean separation.

Dam muscle reserves significantly increased calf muscle metabolic activity (*P* < 0.05; Figure 1B) of calves born to HM dams. We found a significant positive correlation between calf muscle metabolic activity and dam muscle depth at 42 d BEC (*P* < 0.05; r = 0.39). The metabolic activity assay measures the irreversible reduction of NADH, flavin adenine dinucleotide, or cytochrome C using resazurin (Beckett et al., 2019) and the fluorescence produced by resazurin is used as a proxy for metabolic activity. Diet did not affect calf muscle metabolic activity. The mTOR assay revealed no effect of dam muscle reserves or diet on pan-mTOR, but a tendency for dam muscle by diet interaction on phosphorylated mTOR (Figure 1C; *P* = 0.09) with LM-CON calves having lower levels compared with other treatments. HM calves had greater muscle metabolic activity compared with their LM counterparts, which could be linked to greater consumption of AA through metabolic processes leading to lower circulating levels of AA in calf serum. One of the ways mTOR is activated is through AA signaling (Takahara et al., 2020), with His, Leu, and Met being higher in circulation of LM-CON calves. Therefore, we postulate that LM-CON animals may be less efficient at using AA to activate mTOR to support skeletal muscle metabolism and growth.

Calves from LM dams overall had higher circulating Arg, Asp, Glu, His, Ile, Leu, Lys, Met, and Pro (*P* < 0.09; Table 2) compared with calves from HM dams. Serum concentrations of Arg, Asp, His, Lys, and Met decreased when BCVFA was supplemented to LM dams whereas these levels increased when BCVFA was provided to HM dams (muscle × diet; *P* < 0.08). The calves were fasted for 12 h before blood was collected, and the reliance of AA for energy is higher. We had hypothesized that calves born to HM animals would have higher circulating AA because previous reports from our laboratory identified HM cows mobilized more muscle prepartum compared with LM (McCabe et al., 2021), and as such, there could be more circulating AA available to the calf in utero. However, the opposite was identified; thus, we postulated that LM-CON calves had greater circulating AA because their dams were not using AA for muscle protein synthesis until immediately prepartum (Gouveia et al., 2024), and as such, AA could be shunted toward the calf in utero. Circulating AA are challenging to measure in a mature dairy cow because of the complexity of AA dynamics, and thus our postulate was not confirmed at the dam level.

**Table 2:** Serum metabolites of calves at 24 h postnatal born to cows stratified by low muscle (LM) and high muscle (HM) or fed a control (CON) diet or a diet supplemented with branched-chain volatile fatty acids (BCVFA)^1^

Serum metabolite^2^	LM-CON	LM-BCVFA	HM-CON	HM-BCVFA	SEM	Muscle	Diet	Muscle × diet	Correlation with dam muscle depth at 42 d BEC	
									r-value	*P*-value
Immunocrit, %	16.8	13.4	13.3	11.7	2.50	0.34	0.35	0.74	−0.17	0.31
Glucose, mg/dL	182	219	185	196	15.0	0.54	0.15	0.46	−0.08	0.61
Creatinine, ng/μL	8.66	8.97	10.2	10.1	0.79	0.10	0.88	0.82	0.24	0.13
3-MH, ng/μL	3.15	3.10	3.80	3.76	0.50	0.21	0.92	0.99	0.05	0.76
3-MH:creatinine	0.35	0.35	0.41	0.37	0.05	0.48	0.67	0.75	−0.08	0.61
Serum AA, ng/μL										
Ala	80.4	66.1	67.4	72.8	6.30	0.62	0.49	0.13	−0.17	0.30
Arg	48.9^a^	38.1^b^	30.6^b^	38.8^ab^	4.50	0.06	0.78	0.04	−0.29	0.07
Asn	15.9	12.6	12.4	10.7	1.40	0.09	0.11	0.62	−0.33	0.05
Asp	2.73^a^	1.74^b^	1.74^b^	1.95^ab^	0.28	0.17	0.17	0.04	−0.31	0.07
Cys	ND	ND	ND	ND	—	—	—	—	ND	ND
Gln	29.3	33.2	40.7	29.4	5.40	0.48	0.48	0.16	0.01	0.94
Glu	54.0	44.3	39.1	37.7	5.30	0.05	0.30	0.44	−0.33	0.04
Gly	24.5	19.8	23.7	22.0	2.40	0.77	0.20	0.53	−0.10	0.61
His	49.7^a^	37.2^ab^	31.6^b^	38.8^b^	4.10	0.01	0.22	0.08	−0.40	0.01
Ile	12.7^a^	9.61^ab^	7.35^b^	7.14^b^	1.20	<0.01	0.20	0.26	−0.38	0.02
Leu	42.8^a^	34.4^ab^	27.8^b^	24.3^b^	4.30	<0.01	0.18	0.57	−0.39	0.02
Lys	24.3^a^	14.9^ab^	10.5^b^	13.6^ab^	3.00	0.02	0.31	0.05	−0.31	0.06
Met	20.7^a^	14.7^ab^	11.5^b^	16.4^ab^	2.40	0.15	0.84	0.04	−0.18	0.29
Phe	15.9	16.2	12.6	14.1	1.50	0.11	0.59	0.71	−0.33	0.04
Pro	52.1^a^	42.8^ab^	38.7^b^	38.7^b^	4.00	0.04	0.26	0.25	−0.38	0.02
Ser	22.8	22.3	17.2	17.8	2.40	0.04	0.98	0.83	−0.48	<0.01
Thr	18.4	19.7	15.8	16.1	2.10	0.16	0.71	0.81	−0.38	0.02
Trp	18.8	19.2	17.5	14.6	3.00	0.32	0.67	0.58	−0.39	0.02
Tyr	61.6	58.2	54.7	51.9	6.50	0.33	0.65	0.96	−0.21	0.21
Val	36.1	30.5	25.8	24.9	3.40	0.03	0.36	0.52	−0.36	0.03

a,b Values without a common superscript letter differ (*P* ≤ 0.05) when using a Tukey adjustment for mean separation.

1 Treatment means or correlation relationships were determined to be different when *P* ≤ 0.05 and tended to be different when 0.05 < *P* ≤ 0.10. Data are reported as LSM. ND = not detected.

2 3-MH = 3-methylhistidine; 3-MH:creatinine = the ratio of circulating concentrations of 3-MH to creatinine.

Dam prepartum muscle reserves were related to calf birth BW and muscle metabolic activity with HM cows having heavier calves with greater muscle metabolic activity, and higher circulating creatinine levels. Calves born to LM cows had higher circulating levels of multiple AA, but dam prepartum muscle reserves alone had little effect on colostrum AA content. The data indicate that much of the muscle effects appear to be due to in utero exposure because minimal calf responses connect to changes in colostrum. Much of the BCVFA effects are due to muscle by diet interaction, suggesting BCVFA impacts on the calf are dependent upon muscle reserve status of the dam. Further research is needed to investigate the potential genetic merit of higher muscle reserves and the impact on offspring.
